# Lamellae Assembly in Dendritic Spherulites of Poly(l-lactic Acid) Crystallized with Poly(*p*-Vinyl Phenol)

**DOI:** 10.3390/polym10050545

**Published:** 2018-05-18

**Authors:** Nurkhamidah Siti, Eamor M. Woo, Yu-Ting Yeh, Faliang Luo, Vimal Katiyar

**Affiliations:** 1 Department of Chemical Engineering, Institut Teknologi Sepuluh Nopember, Surabaya 60111, Indonesia; nurkhamidah@chem-eng.its.ac.id; 2 Department of Chemical Engineering, National Cheng Kung University, Tainan 701-01, Taiwan; yumi10566@gmail.com; 3 State Key Laboratory of High-Efficiency Coal Utilization and Green Chemical Engineering, Ningxia University, Yinchuan 750021, China; fll@nxu.edu.cn; 4 Department of Chemical Engineering, Indian Institute of Technology Guwahati, Assam 781039, India; vkatiyar@iitg.ernet.in

**Keywords:** poly(l-lactic acid), birefringence, dendrites, ringed spherulites

## Abstract

Lamellar assembly with fractal-patterned growth into dendritic and ringed spherulites of crystallized poly(l-lactic acid) (PLLA), of two molecular weight (MW) grades and crystallized at (temperature of crystallization) *T*_c_ = 120 and 130 °C, respectively, are evaluated using optical and atomic-force microscopies. The results of surface-relief patterns in correlation with interior microscopy analyses in this work strongly indicate that the observed birefringence changes in PLLA polymer dendritic or ringed spherulites (from blue to orange, or to optical extinction) need not be definitely associated with the continuous helix twisting of lamellae; they can be caused by sudden and discontinuous lamellae branching at intersected angles with respect to the original main lamellae, as proven in the case of dendritic and zig-zag rough-ringed spherulites. Intersection angles between the main stalks and branches tend to be governed by polymer crystal lattices; for PLLA, the orthorhombic lattice (α-form) usually gives a 60° angle of branching and hexagonal growth. The branching lamellae then further bend to convex or concave shapes and finally make a 60–90° angle with respect to the main stalks. Such mechanisms are proven to exist in the straight dendritic/striped high-molecular weight (HMW)-PLLA spherulites (*T*_c_ = 120 °C); similar mechanisms also work in circularly ringed (*T*_c_ = 130 °C) HMW-PLLA spherulites.

## 1. Introduction

It is common knowledge that polymer spherulites may grow into compact well-rounded spheres, dendritic patterns, or even periodically ringed entities. Regardless of the final forms, spherulites evolve from an initial crystal aggregation of highly asymmetric nuclei. Spherulites had been generally conceived as symmetric aggregations with lamellae radiating from a common center, but more detailed interior dissection of regions near the nuclei may reveal otherwise. Earlier work by Keith and Padden has suggested that the splaying lamellae in the blend system occur due to the noncrystallizable or low-molecular-weight component being excluded from the growth front, resulting in spherulites with a sheaf-like structure or eye-like region [1,2]. An eye-like region in the center of spherulites is commonly observed in the polymer spherulites of poly(ethylene succinate) (PESu) [3], syndiotactic polystyrene (sPS) [4], and poly[(*R*)-3-hydroxyvalerate] (PHV) [5]. The dramatically different morphologies between the eye-like region and the surrounding lamellae of the spherulites infer that the thermal behavior may also differ. The eye-like region and the surrounding regions (or regular regions) outside it in crystallized sPS have been reported to exhibit different melting temperatures [4], which can be explained by the fact that these two regions might be filled with lamellae of widely different thickness/perfection, and so forth. For PHV, crystals in the eye-like region and surrounding regions have different growth axes, where the lamellae in spherulites apparently grow and twist along the *b*-axis and *a*-axis in the regular and eye-like regions, respectively [5]. In addition, an earlier study has demonstrated that LMW-PLLA (MW ca. 10,000 g/mol) interacting with a H-bonding diluent poly(vinyl phenol) (PVPh) exhibits three different types of crystalline morphology when crystallized at the same *T*_c_ = 120 °C, code-named as Type-1, Type-2, and Type-3 [6]. The three types of PLLA spherulites, crystallized with H-bonding amorphous polymeric diluents, are respectively coded as: Type-1 (hexagonal crystals), Type-2 (dendritic crystals), and Type-3 (Janus-faced combination of Type-1 and Type-2 as a half-and-half “chimera” spherulite).

Dendritic polymer spherulites tend to form either by interacting with strongly interacting diluents (such as PVPh) or by crystallization at relatively high *T*_c_. An interesting comparison between dendritic spherulites of different polymers can be made here. A recent work [7] has revealed that as the strongly interacting amorphous poly(*p*-vinyl phenol) (PVPh at 20 wt %) is added into poly(ethylene oxide) (PEO) and by crystallizing the PEO/PVPh (80/20, *w/w*) mixture at *T*_c_ = 45 °C, dendritic PEO lamellae of alternating birefringence colors are resulted, where the dendritic PEO lamellae intertwine roughly in the radial directions as periodic stripes. The PLLA dendritic lamellae behave similarly as the PEO ones in displaying alternating stripes of birefringence colors in spherulites upon optical microscopy characterization. Optically, they may appear similar; however, upon detailed atomic-force microscopy (AFM) or scanning electron microscopy (SEM) analysis, the lamellae assembly in PEO versus PLLA differs subtly. For PEO dendritic lamellae, the alternating bundles are exactly of the same geometry (of elongated plates, each being 0.2 µm in width and 2–5 µm in length), but they fan out as fractal-tree patterns by mutually intersecting at a ±45° angle, respectively, as they grow away from the radial direction. For the PLLA dendritic lamellae, the lamellae (or bundles) fan out, also as fractal tree-canopy patterns by intersecting at a ±60–90° angle, respectively, as they grow away from the radial direction. PLLA interacts only moderately with atactic poly(methyl methacrylate) (aPMMA). An earlier study has reported that PLLA spherulites, upon crystallization with PMMA, display a Janus-faced half-and-half optical pattern [8]. Detailed AFM/SEM analysis has revealed that one half (along the sheaf-like nuclei) is highly dendritic lamellae, and the other half (perpendicular to the sheaf-like nuclei) is filled with circular-shaped dot-like crystals. The morphologies in the two chimera faces of the PLLA spherulite in the PLLA/PMMA mixture [8] differ dramatically in optical birefringence, growth rates, and crystal size and assembly. 

In addition to PLLA, for many polymers upon melt-crystallization from molten liquids, different types of spherulitic morphology can be resulted depending on the composition of mixtures, crystallization temperature (*T*_c_), sample film thickness, molecular weight, nucleation, and so forth. Isotactic polypropylene (iPP) may exhibit four different types of spherulites (labeled as Type-1, -2, -3, and -4), classified according to the crystal structure, birefringence, banding, and crystallization temperature [9]. For iPP, spherulite forms are also compounded by the presence of lattice polymorphism. It has been reported that there are two possible growth mechanisms leading to the formation of various types of aggregated spherulites: (a) central multidirectional growth, resulting in compact spherulites; and (b) sheaf-like unidirectional growth, resulting in spherulites with an eye-like region in the center of the spherulite [10]. It also has been reported that the four different types of spherulites in iPP have different types of crystal cells. Type-1 and Type-2 iPP spherulites are classified as the α-form with a monoclinic structure, and Type-3 and Type-4 spherulites belong to the β-form with a hexagonal structure [11,12,13,14,15]. Other polymers or polymer blends may also exhibit different types of spherulite morphology at the same *T*_c_, such as in poly(octamethylene terephthalate) (POT) [16], poly(nonamethylene terephthalate) (PNT) [17,18,19], poly(heptamethylene terephthalate) (PHepT) [20,21], poly(vinylidene fluoride) (PVF2) [22,23], and PVF2 blended with a copolymer of styrene and methyl methacrylate (PSMMA) [22,23]. For polymers or blends, different types of spherulitic morphology at the same *T*_c_ are attributed to different crystal cells (lattice forms), for example, in cases of iPP, as found by some investigators. Nevertheless, for PLLA of only the same α-forms, there are three spherulite morphologies coexisting when crystallized at a certain *T*_c_, suggesting that polymorphic crystal forms are not essential factors for multiple types of spherulites.

Crystallization behavior of neat PLLA or its blends with other polymers has been intensively studied, owing to the potential of finding synergistic mixtures for property tailoring. Blending of PLLA with amorphous polymers, such as poly(d,l-lactic acid) (PDLLA) or poly[(*R*,*S*)-3-hydroxybutyrate] (PHB), has been reported to be miscible, and addition of PHB to PLLA tends to decrease the inter-ring spacing of PLLA spherulites [24]. In the case of poly(3-hydroxybutyric acid-*co*-3-hydroxyvaleric acid) (PHBV)/poly(vinyl acetate) (PVAc) blends, the increase of PVAc content in the blends enhances the regularity of ring-banded spherulites of PHBV and the inter-ring spacing significantly decreases [25]. Thus, the phenomenon in the PLLA/PHB blend is quite similar with that of the PHBV/PVAc blend system in that both systems are miscible because of typical polar interactions between the two constituents. By contrast, poly(4-vinyl phenol) (PVPh) contains a pendant –OH group for potential H-bonding interaction with polyesters. PVPh is a nonbiodegradable amorphous polymer with a high glass transition temperature (*T*_g_) that is known to be partially miscible with high-MW PLLA [26]; however, lower molecular weights in PLLA might result in better miscibility in PLLA/PVPh blends, owing to a greater contribution from entropic mixing. Some investigators claimed PLLA/PVPh to be fully miscible [27,28]; however, it should be commented that the molecular weights of PLLA might have influenced the phase behavior, and this should be taken into consideration. Neat, pristine LMW-PLLA only exhibits one type of spherulites upon crystallization at a specific *T*_c_, which is that of well-rounded and compact Maltese-cross spherulites [26]. By contrast, in a homogeneous mixture state with PVPh, the LMW-PLLA/PVPh (70/30, *w/w*) blend exhibits three distinctly different types of spherulites upon crystallization at *T*_c_ = 120 °C [6]. In the LMW-PLLA/PVPh blend, upon crystallization and via H-bonding, PLLA interacts intimately with the –OH group in PVPh [27,28], and was found to display various types of spherulites coexisting when crystallized at the same *T*_c_. In addition, Zhang et al. [29] observed that the PLLA/PVPh (90/10, *w/w*), (80/20, *w/w*), (70/30, *w/w*), and (60/40, *w/w*) blends have spherulitic morphology with a uniformly well-rounded Maltese-cross pattern, showing no unusual lamellar packing in the spherulites. 

Earlier AFM analyses preliminarily revealed that Type-1 and Type-2 PLLA spherulites differ in optical patterns and lamellae assembly, and that the Type-1 PLLA spherulite is mainly composed of dot-like lamellae with a spherical shape, as observed in the center and edge of the spherulites [6]. The lamellar arrangement in Type-2 is mainly circular dot-like crystals in the eye-like region (perpendicular to the sheaf), but with fiber-like crystals in the dendritic portion of the spherulites (along the sheaf). Dendritic PLLA lamellae are not just straight: AFM analysis reveals that all dendritic branches are bent to the counterclockwise direction; however, in such claims, one should realize that imaging from different apparatus might mirror each other. For the polarized-light optical microscopy (POM) apparatus used in this work, the POM images are oppositely reverted (mirror images to each other) to those of AFM. With this adjustment, the bending direction of PLLA in PLLA/PVPh blends is in agreement with the previous investigations on neat PLLA or PLLA blends by Prudhomme et al. [30,31,32,33]. They proposed that as the film thickness approaches nanometers, the optical birefringence of crystallized spherulites can be quite different from that of thicker aggregations. It has been reported that nonbirefringent spherulites in PLLA film samples of nanometer thickness are resulted when the chain axis of the lamellae is parallel to the substrate surface. poly(lactice acid) (PLA) (either the l- or d-form), when confined to ultrathin states, is more likely to transform from aggregated forms to a loose packing of single crystals. It has been observed by Doi et al. [34,35] that the morphology of an ultrathin poly[(*S*)-lactic acid] (PLA) film with thickness of 100 nm changes from spherulitic aggregates to hexagonal crystals at around 150 °C with the increasing of *T*_c_, which is caused by the regime transition from regime II to regime I. Furthermore, by investigating isotactic polystyrene (iPS) with film thickness approaching nanometers (100–300 nm, melt-crystallized at *T*_c_ = 160 °C), periodic rings with optical extinction in the iPS spherulites can be identified, with a proposed depletion draining; and these extinction-banded iPS spherulites are quite different from ringless iPS spherulites with thicker micrometer-thick films [36]. These results in the literature collectively suggest that the film thickness of the crystallizing species serves as one of the key kinetic parameters governing the lamellar assembly to form the final aggregated polymer spherulites.

Studies of the formation of the three types of spherulites in PLLA/PVPh blends focused on their phase behavior, crystallization behavior, and biodegradability; however, the crystalline morphology behavior in this blend system is less studied. This may be due to the addition of PVPh into PLLA greatly restricting the crystallization of PLLA, especially of high molecular weights, in the PLLA/PVPh blends. Until recently, it was first reported that multiple types of spherulites could coexist in PLLA crystallized in blends with strongly interacting PVPh [6]. Responsible factors driving these multiple patterns of lamellae formation and the formation of dramatically different spherulites were preliminarily expounded in some earlier work [6,7,8]; however, the mechanisms of formation of dendritic versus banded PLLA spherulites in various conditions had yet to be justified. Better-refined mechanisms for lamellar assembly into these three distinctly different types of PLLA spherulites coexisting in the same polymer system were probed. One of the main objectives of this continuing work was to analyze how the lamellar assembly patterns can be correlated with the three types of spherulitic optical birefringence. The effects of molecular weight, phase behavior, and intermolecular interactions on the variation of lamellae assembly leading to different fractal-patterned dendrites in PLLA spherulites were among the central focuses.

## 2. Experimental Section

### 2.1. Materials and Preparation

Two grades of low- and high-MW poly(l-lactic acid) (PLLA) were used. Low-molecular-weight poly(l-lactic acid) (coded as LMW-PLLA) was purchased from Polysciences, Inc. (Warrington, PA USA) with a *M*_r_ of 11,000 g/mol and PDI = 1.1, glass transition temperature (*T*_g_) = 45.3 °C and melting temperature (*T*_m_) = 155 °C. For comparison, high-molecular-weight poly(l-lactic acid) (coded as HMW-PLLA) was purchased from Fluka (Milwaukee, WI, USA) with *M*_w_ = 152,000 g/mol and PDI = 1.5. *T*_g_ and *T*_m_ for HMW-PLLA are 59 and 176 °C, respectively. Amorphous polymer, poly(*p*-vinyl phenol) (PVPh), was purchased from Polysciences, Inc. (Warrington, PA, USA) with *M*_w_ = 22,000 g/mol and *T*_g_ = 114 °C.

Samples of LMW-PLLA/PVPh or HMW-PLLA/PVPh blends with two fixed compositions, 70/30 and 50/50 by wt ratios, were prepared by solution blending, using *p*-dioxane as the common solvent with a concentration of 4 wt % (polymers/solvent). Depending on the desired film thickness, an appropriate amount of the well-mixed polymer solution was deposited and uniformly spread on a glass microslide at 45 °C. Subsequently, the solvent in deposited films was allowed to fully evaporate in the ambient atmosphere and temperature. The sample film thickness was kept at ca. 5–10 μm, and within this range of film thickness, past work has proved that the crystallized PLLA morphology remains the same as long as the mixture composition and *T*_c_ are fixed. The microglass slides were used as received, without further solvent or etching treatment of surfaces. For nanometer ultrathin film specimens, treatment of glass surfaces may be necessary, but our films in this work are 5–10 μm thick. The dried films on the microglass slide were crystallized without a top cover. Samples of polymer films (on glass slides) were first heated to melt on a hot stage to a maximum melt temperature (*T*_max_ = 190 °C), held for 2 min for erasing the prior crystals or thermal histories, then rapidly removed to another hot stage preset at a designated isothermal *T*_c_ = 120 or 130 °C until full crystallization (up to 1 h). In this work, unless otherwise indicated, all polymer specimens for analysis were crystallized at these specified *T*_c_s (120 or 130 °C) until full crystallization after holding at *T*_max_ = 190 °C for 2 min.

Note that the objective of this work was to investigate correlations between the optical birefringence patterns with lamellae/crystal assembly in the PLLA polymer spherulites; thus, the sample thickness was kept in the range of 1–10 μm. This film thickness is 100- to 1000-times thicker than a single PLLA lamella; nevertheless, the objective of this work was not dealing with a single lamella of 5–10 nm, which does not display any optical birefringence at all. With aggregated lamellae in a typical polymer spherulite that display their corresponding optical birefringence patterns, interpretations were focused on the summary contributions from the assembly of multiple lamellae/bundles with polycrystalline orientations, as they were not possible for individual lamella.

### 2.2. Apparatus and Procedures

Differential scanning calorimetry (DSC): Thermal transitions of the polymer mixtures were characterized with a differential scanning calorimeter (Perkin-Elmer Corp., Waltham, MA, USA, Diamond model) equipped with a mechanical intracooler for quenching. For *T*_g_ and *T*_m_ determination, each sample was heated to *T*_max_ = 200–220 °C, then rapidly quenched to ambient temperature to initiate scanning. Prior to DSC runs, the temperature and heat of transition of the instrument were calibrated with indium or zinc standards. A continuous nitrogen flow in the DSC sample cell was maintained. DSC was used for evaluating the phase behavior and diagrams of mixtures of PLLA of different MWs with PVPh to construct *T*_g_-composition curves.

Polarized-light optical microscope (POM, Nikon Optiphot-2, Tokyo, Japan), equipped with a Nikon Digital Sight (DS)-U1 camera control system and a microscopic hot stage (Linkam THMS-600, Surrey, UK, with T95 temperature programmer), was used for analyzing the crystalline morphology. 

Atomic-force microscopy (AFM, diCaliber, Veeco Corp., Santa Barbara, CA, USA) investigations were made in an intermittent tapping mode with a silicon tip (*f*_o_ = 70 kHz, *r* = 10 nm) installed. The largest scan range of the instrument was 150 μm × 150 μm, and the scan was kept at 0.5 Hz for overview and zoom-in regions (10 μm × 10 μm. Thin films were deposited on glass slides, and properly crystallized at a specified *T*_c_, with an open face for AFM characterization. AFM measurements were also carried out to determine the height profiles in different crystalline regions of various types of PLLA spherulites in the PLLA/PVPh blend.

## 3. Results and Discussion

**Scheme 1:**
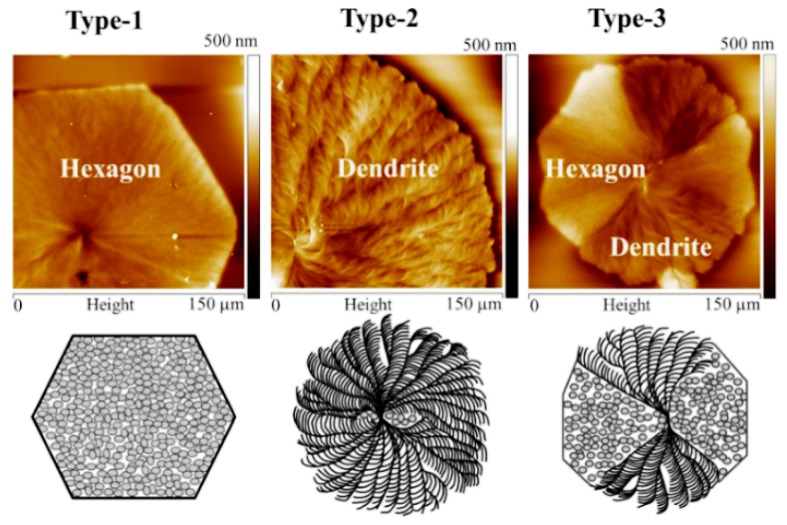
Atomic-force microscopy (AFM) images and schematic patterns for three types of PLLA spherulites coexisting at *T*_c_ = 120 °C, upon crystallization from a LMW-PLLA/PVPh (70/30, *w/w*) mixture. (License Number 4302930696493, copyright permission, reproduced from [6]).

Some illustration is necessary for clarifying the complexity and diversified patterns of polymer spherulites crystallized in various conditions, including in blends with other amorphous polymers. The PLLA spherulites in PLLA/PVPh blends are taken as an example [6]. For direct illustration, the three types of PLLA spherulites are shown as Scheme 1 below (reproduced with permission from [6]), which can be distinguished by their shape, optical sign, and birefringence. When PLLA is crystallized from a 70/30, *w/w* mixture of PLLA/PVPh, three different types of spherulites coexist at the same crystallization temperature (*T*_c_ = 120 °C). An intellectual inquisition is how can the same PLLA polymer crystallize into three dramatically different types of aggregated spherulites simultaneously coexisting, and what are the mechanisms involved? More unique features worthy of probing are what driving forces may be responsible for the coexistence of three uniquely different types of spherulites in PLLA in a single specimen crystallized at the same *T*_c_. Several factors could be proposed, such as PLLA molecular weights, the presence of amorphous PVPh, and crystallization temperature; yet, mechanisms for lamellae self-assembled into multiple types of aggregated spherulites remain unclear.

**Scheme 2:**
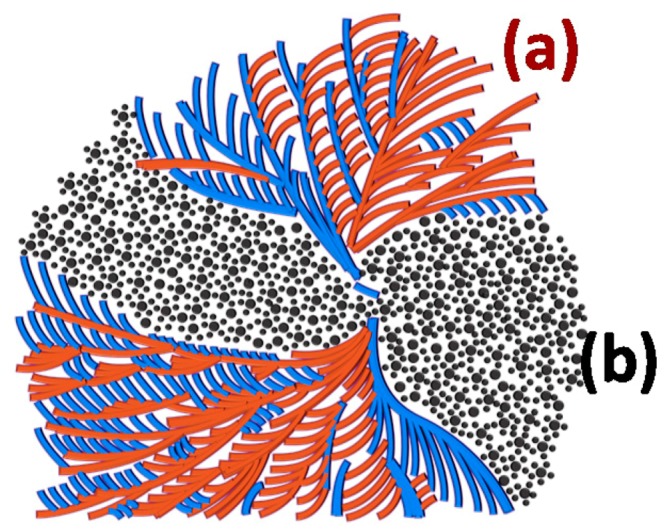
Janus-faced half-and-half spherulites, composed of (**a**) dendritic lamellae in a half portion, and (**b**) circular-dot crystals in the other half portion.

In another earlier work on PLLA spherulites crystallized from PLLA/PMMA blends, a novel approach was taken by dissection into the interior lamellar arrangement of the optically asymmetric spherulites to reveal that large dendritic lamellae, along the direction of the sheaf-like nuclei, grow much faster and periodically bend and branch out as they grow outward, appearing as densely dendritic region(s) with strong birefringence. Oppositely, the nano-sized rod-like lamellae grow much slower in the perpendicular direction, with each rod slanting at a fixed angle to the substrate, appearing as a bivalve-shaped region of weak birefringence. Furthermore, these slanted interior nanorod lamellae emerge to appear as circular-shaped dots on the top surface of the PLLA spherulites. Thus, apparently, PLLA can crystallize into various optical patterns with correspondingly different lamellae assembly mechanisms when it is mixed with diluents of different chemical structures and with different levels of intermolecular interactions. To more clearly and visually aid the understanding of the abovementioned complexity in possible crystal aggregations in polymer spherulites, Scheme 2 depicts an unusual half-and-half Janus-faced morphology of PLLA spherulites crystallized in PLLA/PMMA mixtures, where dendritic lamellae fill one half and dot-like crystals fill the other half of a “Janus-faced” spherulite [8].

The PVPh constituent in the PLLA/PVPh mixtures was intended as an effective diluent for inducing variation of the optical birefringence patterns of the crystallized PLLA spherulites in the mixtures. Systematic variation of *T*_c_, as well as compositions, were done as preliminary work. Too-high *T*_c_ (>130 °C) of crystallization would lead to the gradual distortion of spherulites, causing loss of dendritic patterns and fainter optical birefringence patterns. Variation of compositions of the PLLA/PVPh mixtures was also examined. With too-high PVPh content (e.g., PVPh > 50 wt %) in the PLLA/PVPh mixtures, there would be no PLLA crystallization at all and the crystallized films would appear to be amorphous. For too-high PLLA content in the PLLA/PVPh mixtures, there would be no dendritic patterns in the PLLA spherulites. Only when PLLA content was at an intermediately high composition (70 ± 5 wt %), the crystallized PLLA/PVPh blends would develop dendritic patterns; by contrast, compositions outside this range would not develop dendritic lamellae in the spherulites or would not crystallize at all.

**Figure 1:**
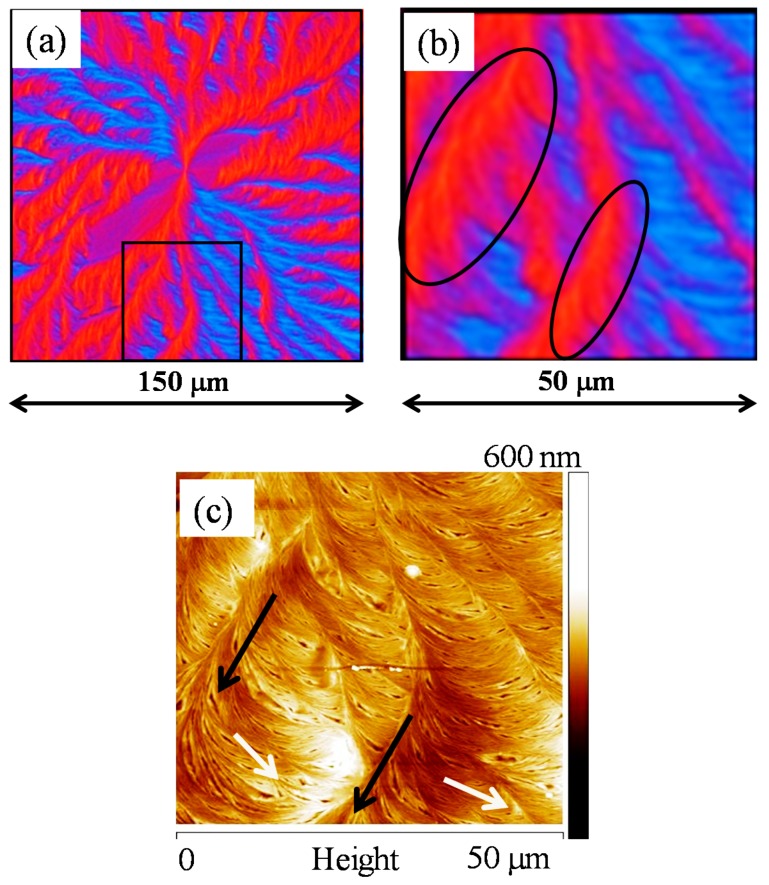
polarized-light optical microscopy (POM) images (flipped) of Low-molecular weight (LMW)-PLLA/PVPh (70/30, *w/w*) melt-crystallized at *T*_c_ = 120 °C. (**a**) Whole spherulites; (**b**) zoom-in of square region of (**a**); and (**c**) the corresponding AFM height image of (**a**).

Phase behavior of the LMW-PLLA/PVPh mixtures was analyzed first. Figure S1 in the Supplementary Materials shows the results of differential scanning calorimetry characterization on LMW-PLLA/PVPh mixtures of various compositions. The POM and DSC results both suggest that the mixtures are nearly miscible, without apparent phase domains, and the *T*_g_-composition curve shows a single *T*_g_ at all compositions investigated in this work. For specifically pinpointing the lamellar assembly to the corresponding optical patterns, POM graphs and AFM images are placed side-by-side for direct comparison to reveal the lamellar bending in the dendritic PLLA/PVPh spherulite. Figure 1 shows POM images of LMW-PLLA/PVPh (70/30, *w/w*) melt-crystallized at *T*_c_ = 120 °C, with the corresponding AFM height image. In this work, we have checked that the bending sense in the POM images represents the correct one of the original morphology, while that in AFM is a mirror image of POM. POM images are mutual mirror images to AFM images, which is to say that the clockwise bending/rotation in POM micrographs would be the counterclockwise one in AFM images, owing to the digital Charge-Coupled Device (CCD) differences in these two apparatus. Thus, the reversion of POM images (or AFM ones) with respect to each other must be taken into consideration in investigating the correlations between the optical birefringence with the actual lamellar assembly and orientations as proved in AFM analyses. To do so, POM images (above) are flipped to reverse the right-hand side to the left-hand side (i.e., mirror-flipping). The flipped POM images would be directly comparable to AFM images, for pinpointing the specific regions. By placing the reverted POM and AFM images directly on a superposition comparison, it is clear to see that the lamellar orientation of the orange color is in the radial direction and the blue color is in the tangential direction, as indicated by arrow marks. This phenomenon can be seen clearly in the orange-color dominated vertical quadrant of spherulites as indicated by the zoomed-in square-marked region of Figure 1a. If the spherulites in the polarized microscopy image are divided into four quadrants, with quadrants 1 and 3 being the orange-color dominated quadrants and quadrants 2 and 4 being the blue-color dominated quadrants, the spherulite is symmetrical, other than the birefringence patterns being divided into two halves. This phenomenon can be seen clearly in the orange-dominated vertical quadrant of spherulites, as indicated in the zoomed-in portion of Figure 1a. On the blue-color-dominated horizontal quadrants, the opposite is true. 

**Figure 2:**
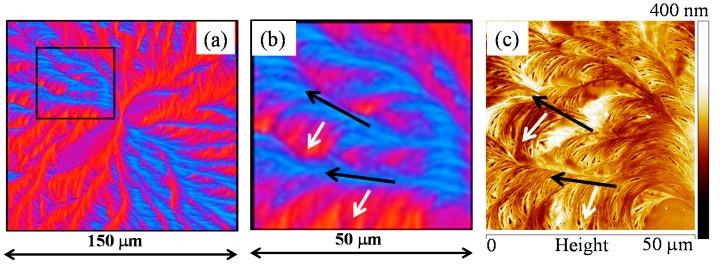
POM images (flipped to mirror image) of LMW-PLLA/PVPh (70/30, *w/w*) melt-crystallized at *T*_c_ = 120 °C. (**a**) Entire dendritic spherulite; (**b**) zoom-in of square region (blue-color-dominated) of (**a**); and (**c**) corresponding AFM height image of (**a**).

Other representative regions of the dendritic PLLA spherulites were analyzed. The blue-color-dominated horizontal quadrants of the POM spherulite image were similarly analyzed using AFM characterization. Figure 2 shows AFM images zoomed in on the square-marked blue-color-dominated regions of the spherulites. By flipping the POM images in the same way as the previous figure and comparing POM (Figure 1a,b) and AFM (Figure 1c) images, it can be observed clearly that the lamellar orientation of the blue-color portion is in the radial direction and that the orange-color portion is in the tangential direction, as indicated by the arrow marks. These two portions of lamellae mutually intersect each other at a 60–90° angle, which respectively displays blue- and orange-color birefringence in the POM micrographs.

The AFM micrographs in Figure 2 show, apparently, that the radiating-lamellar dendrites are composed of straight main stalks and curved branch lamellae. In the orange-color quadrants (vertical) of the spherulite, the lamellar orientation of the orange-color crystals of the main stalks is in the radial direction. Some minor fractions of lamellae bend to the opposite clockwise direction, which shows blue color. In the blue-color quadrants (horizontal quadrants), lamellae are slanted (bent) at 60°; in the orange-color quadrants, bending is similarly at a 60° angle. The 60°-angle of bending is in line with the branching angles in PLLA or poly(ethylene succinate) (PESu) single-crystal packing, as revealed in recent studies [37,38], although the dendritic spherulites are not composed as single crystals, but polycrystalline bundles. The main stalks mostly have lamellar orientation in the radial direction, with branches curving counterclockwise at a 60–90° angle to the main stalks. Note that the lamellae branches do not bend randomly, but tend to bend in the same direction; they actually all bend consistently in a counterclockwise-rotation direction. 

**Figure 3:**
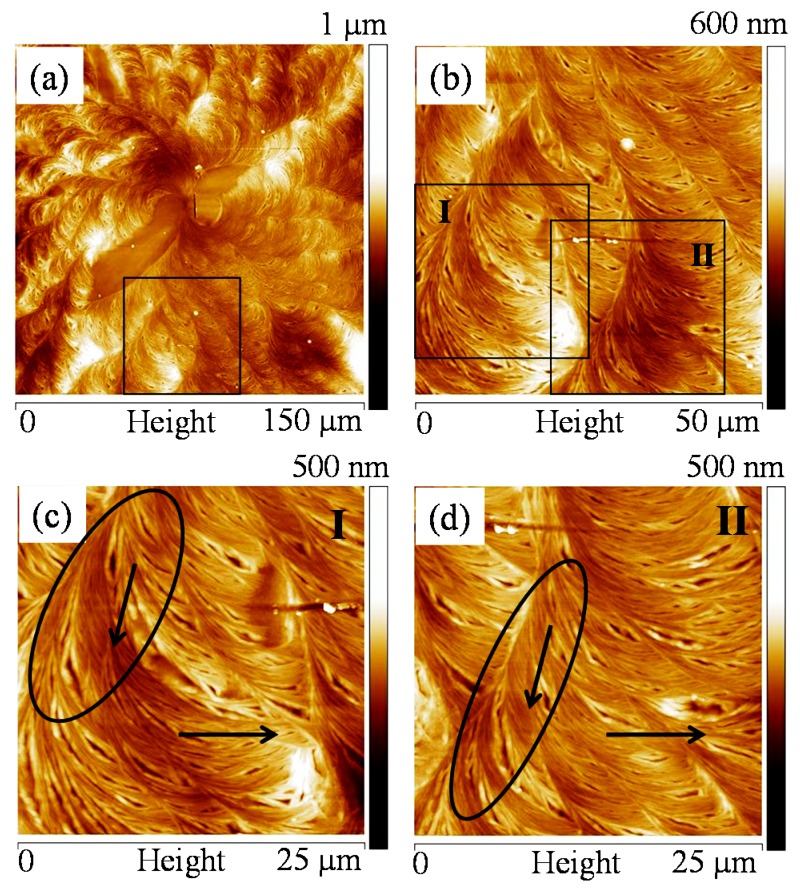
AFM image of LMW-PLLA/PVPh (70/30, *w/w*) melt-crystallized at *T*_c_ = 120 °C. The zoomed-in image of the square-marked region in (**a**) is shown in (**b**). Images (**c**) and (**d**) are of the zoomed-in areas of regions I and II in (**b**), respectively.

Figure 3 shows AFM phase images of an entire PLLA/PVPh spherulite of dendritic (or striped) birefringence patterns and progressive magnifications to increasingly zoomed-in images in Figure 3b–d. Objectives were to identify the lamellae orientation/twisting, bending, or flipping with respect to optical birefringence patterns. For this purpose, specific regions were marked for zoom-in, and comparisons between the optical birefringence and AFM lamellae morphology were made in order to pinpoint the assembly details. The oval-marked regions in AFM images Figure 3c,d correspond to the circled regions in Figure 1b (POM image), showing orange-color birefringence in POM (with a tint plate). One sees that the oval-marked regions in the AFM images display distinctly radial orientations. Numerous fine branches evolve from these radially oriented main lamellae, intersecting at a 60° angle at the beginning, then curving in a counterclockwise direction toward the perpendicular direction, and finally aligning to the tangential direction of the dendritic spherulites. Such curving trends of branches are visible in the POM images as the orange-color stripes, while the radial straight stalks are seen as the blue-color stripes in the POM images. 

This is direct evidence that perpendicular intersection of lamellae in either dendritic or ring-banded spherulites is a common habit of crystal packing. The dendritic spherulites are also characterized with bundles of branches evolving from main stalks and bending at a 90° angle as they grow to fill the increasing space of the spherulites. Note that the AFM images clearly reveal that the ends of these tangentially oriented branches come to be interfaced with the neighboring radial lamellae at a 90° angle; that is, the fine lamellar branches from the radial lamellae appear to evolve only in a counterclockwise direct in one side, but not from both sides of the radial ones.

By these direct comparisons of the AFM morphology and POM birefringence patterns, it can be concluded that the lamellar orientation of crystals of orange-color birefringence in POM images is in the radial direction and that of the other crystals of blue-color birefringence is in the tangential direction, as indicated by the arrow marks in the AFM images. These remarks apply only to the square-marked regions of spherulites in Figure 1a. When different regions of the dendritic spherulites are marked for analysis, the opposite results may apply; but when a specific region of the same quadrants of spherulites is chosen, lamellae of opposite birefringence colors (orange/blue) indicate that they differ by intersection at a 90° angle. These AFM and POM analyses on the striped/dendritic PLLA/PVPh spherulites reveal that there is no need to helix-twist continuously from a flat-on angle gradually to an edge-on angle to cause the alternating optical birefringence changes. 

**Figure 4:**
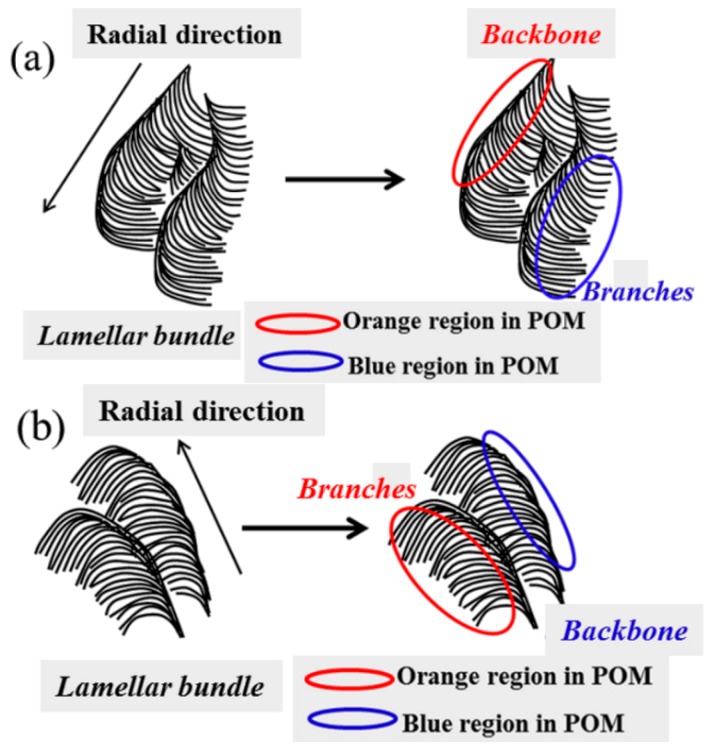
Schemes illustrating correlations between the POM color stripes and AFM lamellar assembly with perpendicularly intersecting branches in two opposite colors of birefringence: (**a**) orange-color stripes and (**b**) blue-color stripes of spherulites.

It is apparent that in the dendritic PLLA spherulites, there are two orange-color quadrants and two blue-color ones. This is similar to most spherulites. However, the difference is that in the quadrants dominated by one birefringence color, there are thinner stripes of the opposite color that are sandwiched. In addition, the lamellae in the dendritic PLLA spherulite are not straightly and radially pointing outward, but all curved to a counterclockwise bending direction. Such bending of the branched lamellae from the main stalks is vividly revealed in the AFM images, as earlier discussed. Figure 4 shows schemes illustrating correlation between the optical color stripes (POM) and lamellae assembly (AFM), with perpendicularly intersecting branches in the orange-color lamellae versus the blue-color lamellae of the dendritic PLLA spherulites. These schemes for illustrating the lamellae assembly and orientation are based on the POM and AFM results already discussed in the previous figures. In the orange-dominated vertical quadrant, the lamellar orientation of orange-color corresponding to the backbone region is in the radial direction. However, the branches correspond to the blue birefringence color and their lamellar orientation is in the clockwise direction. Contrarily, in the blue-dominated quadrant of the PLLA spherulite, the backbone lamellae correspond to the blue color, and are oriented straightly in the radial direction, with the orange-color branches bent/curved in the clockwise direction. From these AFM results and illustrative schematic graphs, it can be concluded that all branches curve to the same clockwise direction, and they intersect at a 60° angle with the main stalk initially, but bend collectively to the same direction as they extend out to make a roughly 90° angle with the main stalks.

**Figure 5:**
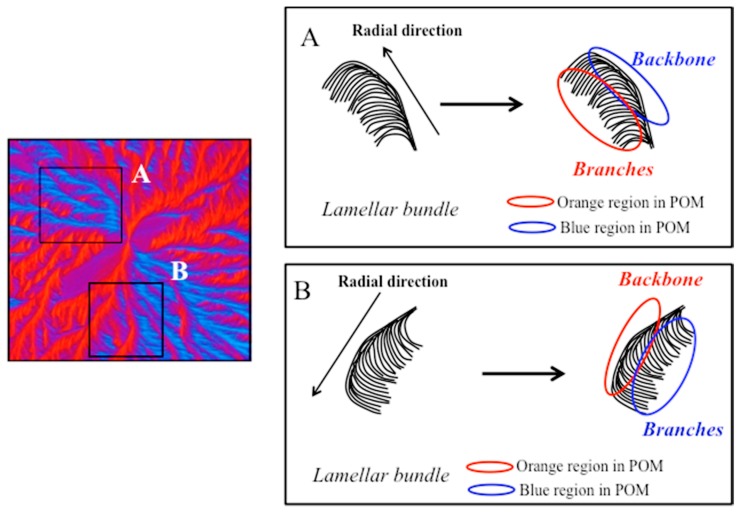
POM image (**left**) and scheme (**right**) for lamellae in dendritic LMW-PLLA/PVPh (70/30, *w/w*) spherulites melt-crystallized at *T*_c_ = 120 °C.

To more emphatically illustrate the correlation between the lamellae orientation revealed in the AFM results and optical birefringence patterns in the POM results for the dendritic PLLA spherulites, Figure 5 shows the direct correlation between the color stripes in the POM images and the lamella assembly with perpendicularly intersecting branches revealed in AFM images, as discussed from earlier figures. The POM graph of the PLLA/PVPh blend is characterized by orange-color-dominated lamellae in the vertical quadrants of the spherulite (a spherulite being dissected into four quadrants by optical Maltese-cross) and blue-color-dominated lamellae in the horizontal quadrant. Note that the optical birefringence of this PLLA spherulite is of an “unusual” type, meaning that the Maltese-cross is tilted at certain angles from the “usual” perpendicular-horizontal position. The schematic drawings for the assembly/orientation of the dendritic lamellae in Figure 5 are based on the AFM results discussed earlier. The correlation clearly points out that the lamellae evolve and grow in a fractal pattern, and that those in the radial direction display an opposite birefringence color from the tangential branches curving/intersecting at a 60–90° angle with the radial ones.

In addition to the morphology behavior in LMW-PLLA, high-molecular-weight PLLA specimens were similarly prepared and analyzed. The phase behavior of HMW-PLLA/PVPh mixtures was analyzed similarly to that of LMW-PLLA/PVPh ones. Figure S2 in the Supplementary Materials shows the results of differential scanning calorimetry (DSC) on HMW-PLLA/PVPh mixtures of various compositions. The *T*_g_-composition curve shows a discontinuity cusp in the curve, indicating possibly partial miscibility with a small-scale phase separation.

**Figure 6:**
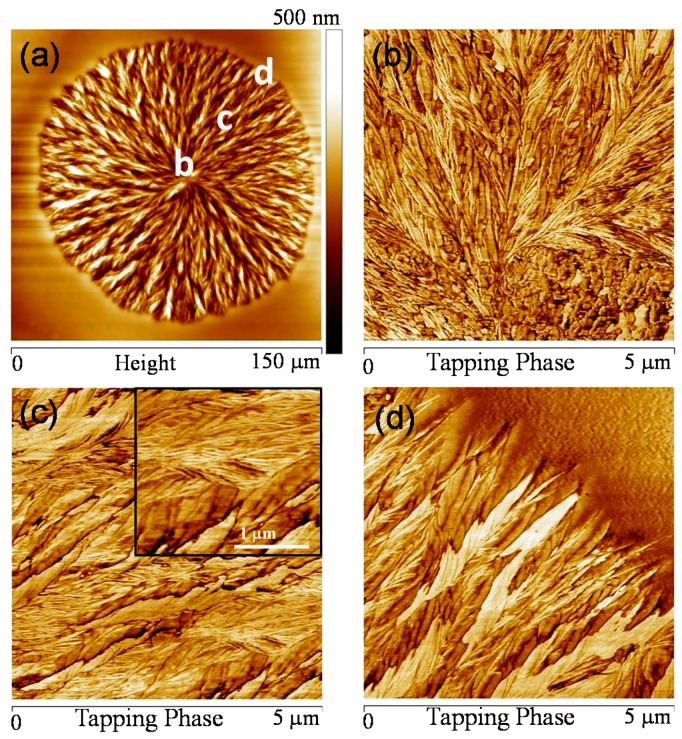
AFM images for HMW-PLLA/PVPh (70/30, *w/w*) blends crystallized at *T*_c_ = 120 °C: (**a**) entire spherulites; (**b**–**d**) zoomed-in images of the corresponding regions (**b**–**d)** indicated in (**a**), respectively, for views at greater magnifications.

HMW-PLLA/PVPh (70/30, *w/w*) samples were crystallized at *T*_c_ = 120 or 130 °C, that is, thermally treated same as the LMW-PLLA/PVPh blend. The spherulites and lamellae in the HMW-PLLA/PVPh blend, as revealed by POM patterns, were further characterized using AFM for revealing more detailed lamellar assembly in the spherulites. The dendritic POM patterns of HMW-PLLA/PVPh (70/30, *w/w*) are similar to those of LMW-PLLA/PVPh, and will not be duplicated here. Figure 6 shows AFM images for the HMW-PLLA/PVPh (70/30, *w/w*) blend crystallized at *T*_c_ = 120 °C and the corresponding zoomed-in micrographs at larger magnifications. Along a radial line, marked as (b), (c), and (d), respectively, are the nucleus, mid-way, and periphery of a spherulite. Figure 6b shows the nucleus region, where fibrous dendritic lamellae radiate out along the nucleus-sheaf direction (a +45° angle along the diagonal line of graph), and bend to splay/fan out with multiple branches. Perpendicular to the nucleus-sheaf direction, the spherulite is filled with irregular dot-like grainy crystals. Figure 6c shows the AFM zoom-in of the mid-way region of the spherulite. The dendrites are filled with bundles of branches, where some are fibrous edge-on-oriented, but others are twisted irregularly to various flat-on orientations. Figure 6d shows the AFM zoom-in of the peripheral region of the dendritic spherulite, where the lamellae are mingled with roughly equal proportions of flat-on platelets and edge-on fibrous lamellae. They then taper to thin needle-like ends upon reaching the periphery. From the AFM phase images of points (b), (c), and (d) of Figure 6a, it can be concluded that this compact spherulite is arranged with alternating edge-on and flat-on lamellae in a radial direction, and that the distribution of these two types of lamellae is random. Note that for the HMW-PLLA/PVPh blend, the compactness of the dendrites is higher than that of LMW-PLLA/PVPh; thus, it is much harder to distinguish the bending direction of the tangential branches from the radial lamellae. However, fractal patterns with tree-canopy-like growth of branches periodically from the nuclei centers all the way to the periphery are apparent in all the spherulites.

### 3.1. Transition from Dendritic to Circularly Ringed PLLA Spherulites at Higher T_c_

**Figure 7:**
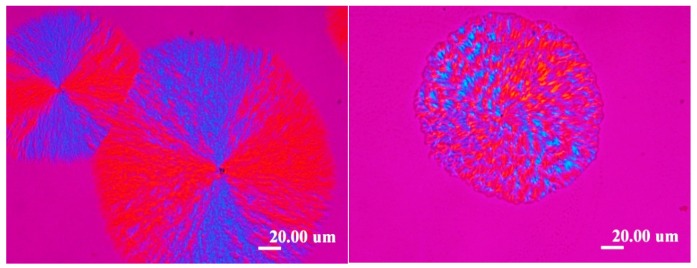
POM micrographs of the HMW-PLLA/PVPh (70/30, *w/w*) blend melt-crystallized at (**left**) *T*_c_ = 120 °C (**right**) *T*_c_ = 130 °C, showing only a single type of spherulite at each *T*_c_. (License Number 4302930696493, copyright: Figure 11 of [6]).

It has been proved earlier that only a single type of spherulite is present in the HMW-PLLA/PVPH (70/30, *w/w*) blend when crystallized at a certain *T*_c_; that is, multiple types of spherulites could not be found in the HMW/PLLA/PVPh blend at any *T*_c_, and this feature is a significantly different morphology behavior from that of the LMW/PVPh blend, which is able to show three different types of PLLA spherulites at a selected *T*_c_. Figure 7 (reproduced with permission from [6]) shows PLLA spherulites of two different types: dendritic (left) versus roughly circular-ringed (right) PLLA spherulites, respectively, crystallized at *T*_c_ = 120 and 130 °C. As discussed, PLLA/PVPh (70/30, *w/w*) crystallized at the lower *T*_c_ = 120 °C exhibits straight-dendrite spherulites (hexagon-shaped); however, at *T*_c_ = 130 °C, the same HMW-PLLA/PVPh (70/30, *w/w*) blend is crystallized into circular zig-zag ringed spherulites (circular shaped), as opposed to the straight-dendrite ones formed at 120 °C-crystallization. The mechanisms of transition from mostly dendritic spherulites to mainly circular ringed spherulites with only a 10 °C increase in the crystallization temperature demands some analyses for explanation. Later on, we will show that nuclei geometries differ for these two types of spherulites, and from the very beginning, these two nuclei types simultaneously exist at either 120 or 130 °C, but the relative fractions of these two types of nuclei may change with *T*_c_. The different fractions of the two types of nuclei in the PLLA/PVPh mixture at different *T*_c_s eventually, upon full crystallization, leads to the dendritic spherulites dominating over the circular zig-zig ringed ones at *T*_c_ = 120 °C; vice versa, at the higher *T*_c_ = 130 °C, the circular zig-zig ringed spherulites dominate.

**Figure 8:**
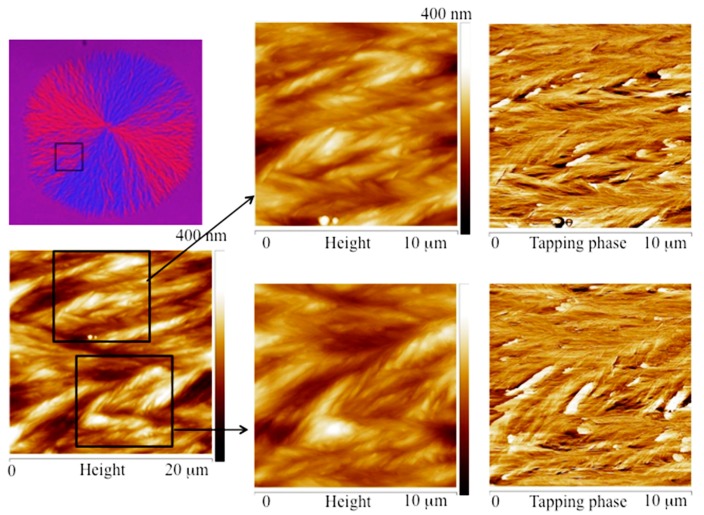
POM and AFM height and phase images of the HMW-PLLA/PVPh (70/30, *w/w*) dendritic spherulite crystallized at *T*_c_ = 120 °C.

Figure 8 shows AFM images zoomed-in on specific regions of the dendritic HMW-PLLA/PVPh (70/30, *w/w*) spherulite that was crystallized at *T*_c_ = 120 °C. The POM image of the same sample is inserted as an inset of the figure to enable appreciation of the birefringence patterns. The POM and AFM images both suggest that the dendrites are not really straight, but randomly and slightly twisted/curved as they radiate out. The AFM images reveal that the dendritic lamellae display a wheat-straw pattern, with main stalks orienting at a 60° angle to the branching finer lamellae. The wheat-straw-like lamellae fan out from the nuclei center with a random fractal assembly to fill the ever-expanding space until the final dendritic spherulite is formed.

Apparently, a lower *T*_c_ at or below 120 °C favors the formation of straight dendritic PLLA spherulites, while the higher *T*_c_ = 130 °C favors the formation of rough-ringed spherulites (i.e., zig-zag banded). Lamellar arrangement in the HMW-PLLA/PVPh blend crystallized at *T*_c_ = 120 °C versus 130 °C is compared. For the dendritic PLLA spherulites at *T*_c_ = 120 °C, both needle-like and platelet lamellae are arranged as fractal tree-like patterns in the radial direction. By contrast, for the rough-ringed PLLA spherulites at *T*_c_ = 130 °C, the dual optical colors (blue/orange) are composed of alternating wide and narrow circular bands. AFM images reveal that the rough-ringed PLLA spherulites are packed by alternating bands of fibrous needle-like lamellae (in the higher narrow band) and fish-scale-like platelet lamellae (in the lower wide band). Nevertheless, these two bands (narrow and wide bands) cannot be conceived as a gradual and continuous twist of one to another; the lamellae in these two bands have completely different geometries and are discrete, discontinuous, and not interconnected. 

**Figure 9:**
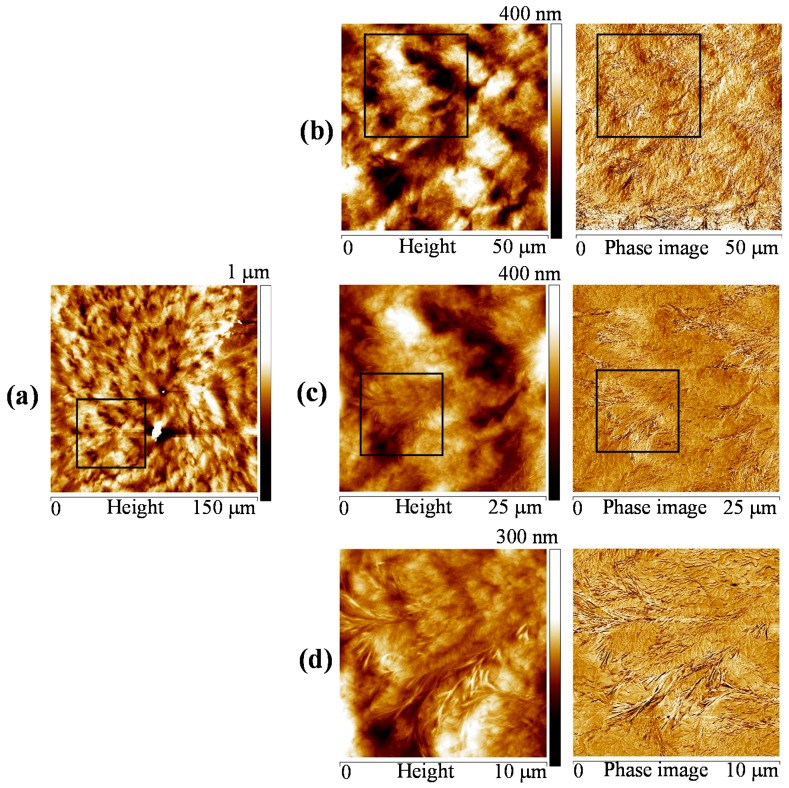
AFM images of a HMW-PLLA/PVPh (70/30, *w/w*) zig-zag rough-ringed spherulite crystallized at *T*_c_ = 130 °C. (**b**) Zoomed-in image of the square-boxed area in (**a**); (**c**) zoomed-in image of the square-boxed area in (**b**); (**d**) zoomed-in image of the square-boxed area in (**c**).

The lamellae in the HMW-PLLA/PVPh rough-ringed spherulites (with zig-zag ring bands) instead of straight-dendrite patterns, as revealed by POM, were further characterized using AFM for analyzing in detail the lamellar assembly in the ringed PLLA spherulites. The AFM images in Figure 9 show that there are “dual bands” in the zig-zag ring-banded PLLA spherulites (*T*_c_ = 130 °C). There are several features worthy of in-depth discussion. There are irregular yet distinctly “dual-color bands” in the spherulites. The dual-color band is structured to comprise a wide and narrow band as one entity, with the wide band as the valley and the narrow one as the ridge. When optically viewed in polarized light with a tint plate (lambda plate), the lamellae of the wide band (lower valley) display a birefringence color; conversely, the narrow band (higher ridge) is always composed by needle-like lamellae, where the optical color of the narrow bands is opposite to that of the wide bands when viewed in POM with tint plates.

**Figure 10:**
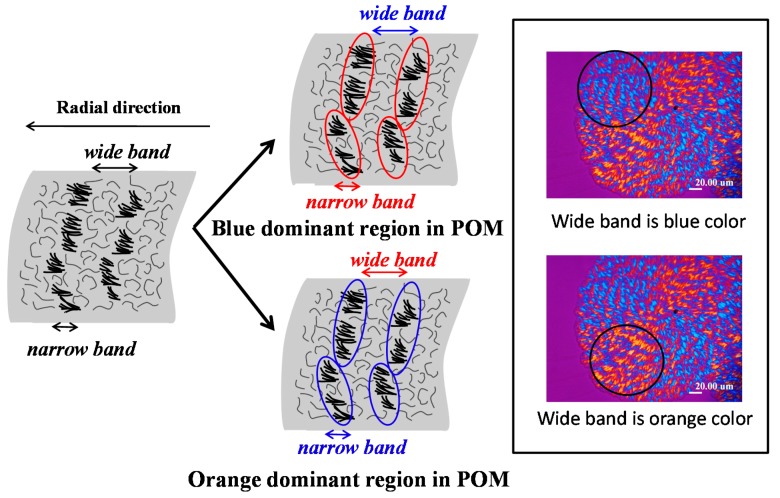
Schemes of lamellae assembly in correlation with the POM birefringence patterns for zig-zag ring bands for lamellar assembly in the zig-zag ringed spherulites of the HMW-PLLA/PVPh (70/30, *w/w*) blend crystallized at *T*_c_ = 130 °C.

Furthermore, Figure 10 shows the scheme for lamellar assembly with the POM micrograph of a zig-zag ringed PLLA spherulite, with optically alternating wide bands and narrow bands in the zig-zag ringed spherulites. As discussed earlier, the two bands in the zig-zag ring-banded PLLA spherulites are arranged as platelets and needle-like lamellae, respectively. The schemes illustrate that the wide band (lower valley) is always composed by platelet lamellae, and the narrow band (higher ridge) is always composed by needle-like lamellae, where the birefringence color of the bands associated with the needle-like lamellae is opposite to that of the platelet crystals.

**Figure 11:**
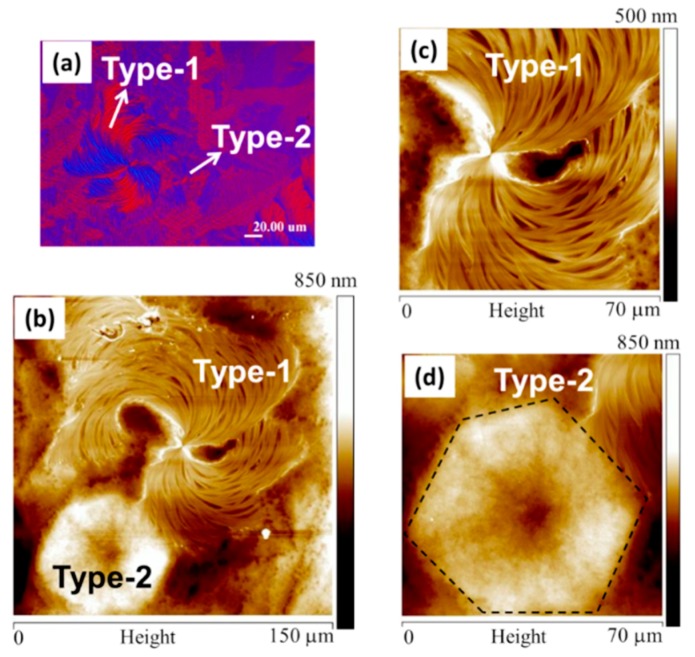
(**a**) POM micrograph, (**b**,**c**) AFM images for Type-1 (dendritic), and (**d**) AFM image for Type-2 PLLA (hexagon) spherulites in HMW-PLLA/PVPh blends (50/50, *w/w*) crystallized at *T*_c_ = 120 °C (after holding at *T*_max_ = 190 °C).

As discussed, at PLLA/PVPh 70/30 composition, PLLA crystallizes only to a single type of spherulite at a certain *T*_c_ (either straight dendrite at *T*_c_ = 120 °C or zig-zag ringed at *T*_c_ = 130 °C); however, at PLLA/PVPh = 50/50, *w/w*. PLLA is again able to crystallize to multiple types of spherulites, just like the LMW-PLLA/PVPh system. The amorphous PVPh in the blend was increased from 30 to 50 wt %, and the effect of composition on PLLA morphology was further probed. Figure 11 shows POM and AFM images for PLLA spherulites at *T*_c_ = 120 °C. Note that in this HMW-PLLA/PVPh (50/50, *w/w*) blend crystallized at the specific temperature of *T*_c_ = 120 °C, there are also two different PLLA spherulites coexisting: (Type-1) round-shaped dendritic types with fibrous lamellae displaying counterclockwise Archimedean spiraling from both ends of the nucleus sheaf; and (Type-2) hexagon-shaped spherulites packed with pebble-like (or platelet) crystals. Higher diluent PVPh content (50 wt %) in the PLLA/PVPh mixture tends to induce multiple types of PLLA spherulites coexisting when crystallized at a specific *T*_c_. This might be attributed to slight differences of localized heterogeneity in the phase domains, leading to a nonuniform strength of interactions between the two components.

**Figure 12:**
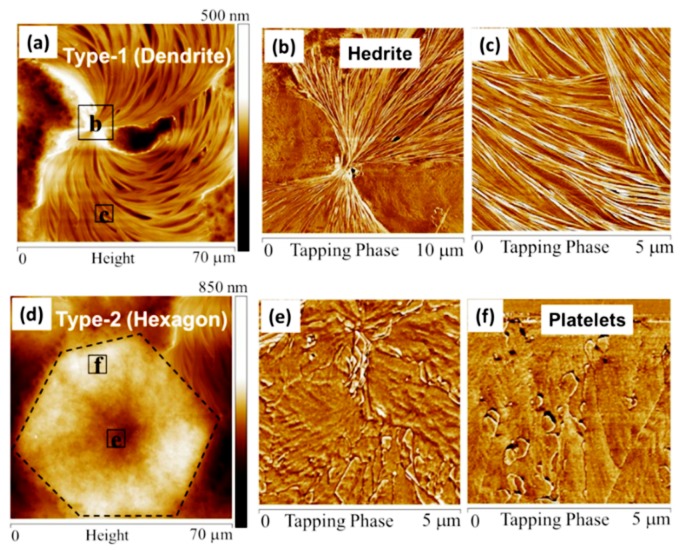
(**a**) AFM image for entire Type-1 dendritic spherulite, (**b**,**c**) zoom-in images for squared-regions-b,c of Type-1 PLLA dendritic spherulite in HMW-PLLA/PVPh blend (50/50, *w/w*) and (**d**) AFM for entire Type-2 (hexagon shape) spherulite; (**e**,**f**) zoom-in images for squared-regions-e,f of Type-e PLLA hexagon spherulite in HMW-PLLA/PVPh blend (50/50, *w/w*) crystallized at *T*_c_ = 120 °C (after holding at *T*_max_ =190 °C).

Specific spots of the Type-1 and Type-2 PLLA spherulites were zoomed in on to obtain greater magnification of the finer details in the morphology. The top diagrams of Figure 12 show Spot-1 (near the nucleus) and Spot-2 (near the dendrite bundles) of the fibrous dendritic Type-1 PLLA spherulites, where AFM zoom-in images are shown for Type-1 PLLA spherulites (fibrous lamellae with dendritic patterns) in the PLLA/PVPh (50/50, *w/w*) blends. Near the nucleus, the nucleus sheaf displays a distinct hedrite shape. In Spot-2, the lamellae are fibrous, similar to bundled wheat straws, and all with counterclockwise spiraling. Similarly, the bottom images of Figure 12 show Spot-1 and Spot-2 of the hexagon-shaped Type-2 PLLA spherulites, where AFM zoom-in images of Spot-1 and Spot-2 are shown for Type-2 PLLA spherulites (hexagon-shape) in the PLLA/PVPh (50/50, *w/w*) blends. Spot-1 is near the nucleus, where morphology is characterized by jammed circular platelets (diameter < 1 μm). Spot-2 is near the edge of the hexagon-shaped spherulites, where the platelets of the crystals are seen to align as a mosaic pattern across the hexagon-shaped PLLA dendritic spherulites.

### 3.2. Growth from Nuclei to Fully Aggregated Spherulites

**Figure 13:**
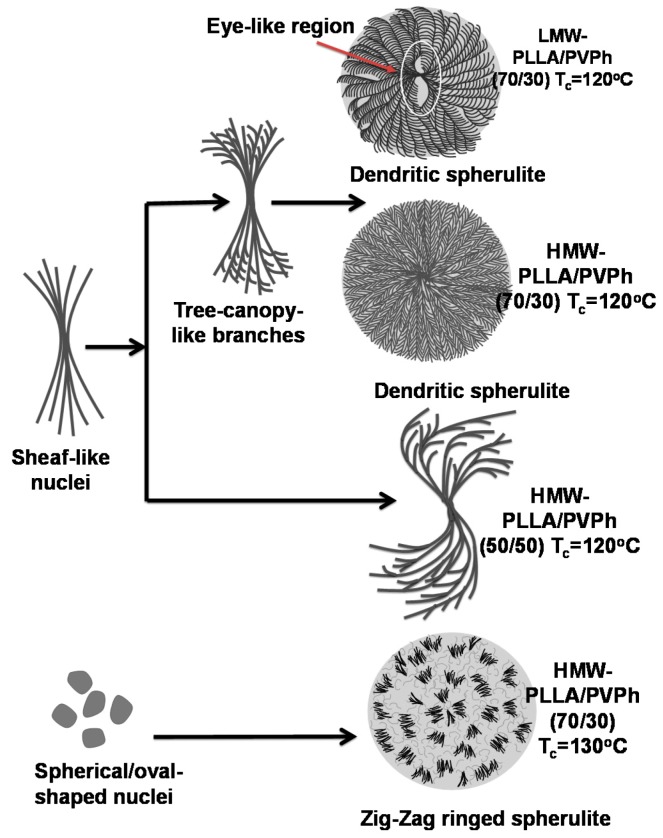
Composition- and *T*_c_-sensitive morphology with three kinds of dendritic spherulites in LMW- or HMW-PLLA/PVPh blends of two compositions (70/30, 50/50, *w/w*) all crystallized at *T*_c_ = 120 °C (after holding at *T*_max_ = 190 °C). At the higher *T*_c_ = 130 °C, the nuclei shapes are different, leading to zig-zag ringed spherulites.

The above AFM analyses have disclosed the main features of the dendritic PLLA spherulites of either HMW- or LMW-PLLA by crystallization from PLLA/PVPh (70/30 or 50/50, *w/w*) blends, where PVPh was used as a polymeric diluent to strongly interact with PLLA via proven H-bonding in the miscible domains. Figure 13 shows three possible growth schemes originating from a sheaf-like bundle of nuclei and gradual evolution into three major types of PLLA dendritic spherulites, differing in the compactness of the lamellae/branches in the filled spherulites. Molecular-weight dependence of dendritic spherulites in the PLLA/PVPh (70/30, *w/w*) blend can be observed at *T*_c_ = 120 °C. For the HMW-PLLA/PVPh blend, the PLLA spherulite exhibits a straight-dendrite pattern and shows more compact lamellae arrangement in comparison to that of the LMW-PLLA/PVPh counterpart. In the LMW-PLLA/PVPh blend, a bundle of branches evolves from the main stalk and bends at a 60–90° angle as they grow to form spherulites. Both LMW-PLLA/PVPh and HMW-PLLA/PVPh blends show a fractal pattern with tree-canopy-like growth of branches from the nuclei to the periphery of the spherulites (see schemes in Figure 13). A composition-dependent morphology of dendritic spherulites in the HMW-PLLA/PVPh blend specimens (70/30 and 50/50, *w/w*) can be all crystallized at *T*_c_ = 120 °C (after holding at *T*_max_ = 190 °C). Two distinctly different types (Type-1 and Type-2) of spherulites coexist when crystallized in the partially miscible HMW-PLLA/PVPh (50/50, *w/w*) blend, which suggests that it is likely that Type-1 spherulites are more influenced by the presence of H-bonding during crystallization (in the miscible domains), while Type-2 (hexagonal type) spherulites are not influenced by this as much (in the less miscible domains). The AFM morphology of Type-1 and Type-2 spherulites was discussed earlier from Figure 12. This is evidenced by the fact that the latter type can also be seen in crystallized neat PLLA (i.e., undiluted by PVPh). Depending on the molecular weight of PLLA and PVPh content (30 or 50 wt %) in the PLLA/PVPh blend, there are three main morphological features in the dendritic PLLA spherulites (at *T*_c_ = 120 °C). Type-1 dendritic spherulites are present in PLLA/PVPh mixtures of all molecular weights (low- and high-molecular-weight PLLA) and compositions (70/30, 50/50, *w/w*), but Type-2 spherulites (hexagon-shaped) are seen only in LMW-PLLA/PVPh, as shown in [6], and in a highly diluted blend (i.e., 50/50, *w/w*). All Type-1 spherulites are crystallized in the same way, from the sheaf-like nuclei, by splaying out from the two ends of the nucleus, but the fibrous lamellae grow quickly with fractal branching to fill out the spherulite into a well-rounded sphere. All three morphologies of the PLLA spherulites in the PLLA/PVPh blends are dendritic with a consistent bending direction of the fibrous lamellae. The morphologies differ in the shapes and birefringence patterns of the spherulites and the extent of dendrites and branches, whose top-surface lamellae assembly is well analyzed by the AFM analyses, as discussed earlier. At lower PVPh contents (such as 30 wt %), the dendritic spherulites are highly filled to a rounded-spherical shape and the compactness is different depending on the molecular weight of PLLA. With PVPh content = 50 wt % in the PLLA/PVPh mixtures, the lamellae/crystals no longer assume a spherical shape, but take an elongated and bent hedrite pattern, which largely resembles the original sheaf-like nuclei, except for the prominent bending of splaying lamellae at the two ends. Thus, the effects of dilution by the amorphous PVPh contents on the PLLA lamellae assembly are quite prominent.

By dramatic contrast, the bottom scheme of Figure 13 shows that for HMW-PLLA/PVPh (70/30, *w/w*) at the higher *T*_c_ = 130 °C, the nuclei shapes are different, leading to zig-zag banded spherulites, and not dendritic spherulites at all. The HMW-PLLA/PVPh (70/30, *w/w*) blend crystallized at *T*_c_ = 130 °C exhibits zig-zag ringed spherulites instead of dendritic spherulites, where the dual optical colors (blue/orange) are respectively composed of fibrous needle-like lamellae in the narrow band as the ridge and fish-scale-like platelet lamellae in the wide band as the valley of the ringed spherulite. The results suggest that the degree of supercooling, or *T*_c_, tends to determine the initial shapes of nuclei, which in turn govern the final spherulite morphology. The abovementioned means of lamellae assembly into the final aggregated spherulites are all shown summarily in the flow-chart schemes of Figure 13.

Note that the dendritic types of polymer spherulites and ring-banded ones are on two opposite spectra of spherulite patterns. Although the investigation of crack formation in dendritic spherulites is not an aim of this work, for comparison purposes, it is worthwhile to mention an earlier work on the cracking of ring-banded versus ringless spherulites, including that in ring-banded or dendritic PLLA spherulites. In an earlier review article [39], we surveyed the plausible formation mechanisms of cracks of mainly circularly ring-banded polymer spherulites. One main conclusion in that review article is that with only some rare exceptions, most of the crack patterns are highly guided by, and correlated with, the lamellar assembly in either ring-banded spherulites or ringless spherulites. The cracks in dendritic spherulites of polymers always tend to be roughly random and not regularly circular or with spiral shapes such as those seen in the circularly ring-banded spherulites. 

## 4. Conclusions

Two grades of PLLA of different MW were examined in terms of lamellae assembly mechanisms in their dendritic and ringed spherulites at *T*_c_ = 120 and 130 °C, respectively, in two different PLLA/PVPh compositions (70/30 vs. 50/50, *w/w*). Mechanisms of the coexistence of multiple types of PLLA spherulites in the crystallized PLLA/PVPh blends were interpreted. Although PLLA polymer spherulites grow from similar sheaf-like nuclei at very beginning, the immediate geometric shapes at both ends of the nuclei, as influenced by kinetic factors such as *T*_c_ and the presence of diluents during crystallization, governed growth into compact, dendritic, or ringed LMW-PLLA spherulites; or the simultaneous presence of all three types in varying fractions. PLLA (HMW-grade) spherulites are shown to change from their original dendritic form at *T*_c_ = 120 °C to zig-zag ringed spherulites at *T*_c_ = 130 °C.

From these facts, it is difficult and unproven to suggest that the conventionally proposed chain-folding stresses could differ significantly from being nonexistent (for dendritic) to being in full-scale existence (for ringed spherulites) within a narrow 10 °C *T*_c_, in order to pack the lamellae into the dendritic types versus the ring-banded types of PLLA spherulites. There must be alternative mechanisms, as stated above. Furthermore, subsequent lamellae splaying and branching during growth can also be influenced by the above kinetic factors, making the aggregation of lamellae into spherulites a very complex process; yet, the final forms are statistically the same or predictably similar as long as the crystallization parameters are controlled. Crystal assembly in either dendritic or ringed spherulites (from blue to orange, or to optical extinction) need not be definitely associated with the continuous helix twisting of lamellae; they can be caused by sudden and discontinuous lamellae branching at intersected angles (60–90°) with respect to the original main lamellae stalks, as proven in the case of dendritic and zig-zag rough-ringed PLLA spherulites.

## Supplementary Materials

The supplementary materials are available online at http://www.mdpi.com/2073-4360/10/5/545/s1.
