# Supplementary material for: Lamellae Assembly in Dendritic Spherulites of Poly(l-lactic Acid) Crystallized with Poly(p-Vinyl Phenol)
Source: Polymers (Basel). 2018 May 18;10(5):545. doi: 10.3390/polym10050545 (PMC6415417; doi:10.3390/polym10050545)
Supplement: Supplementary file 1 [file polymers-10-00545-s001.pdf]

polymers

MDPI

Lamellae Assembly in Dendritic Spherulites of Poly(L-lactic Acid) Crystallized with Poly(*p*-Vinyl Phenol)

Nurkhamidah Siti [1], Eamor M. Woo [2,*], Yu-Ting Yeh [2], Faliang Luo [3] and Vimal Katiyar [4]

1 Department of Chemical Engineering, Institut Teknologi Sepuluh Nopember, Surabaya 60111, Indonesia; nurkhamidah@chem-eng.its.ac.id
2 Department of Chemical Engineering, National Cheng Kung University, Tainan 701-01, Taiwan; yumi10566@gmail.com
3 State Key Laboratory of High-Efficiency Coal Utilization and Green Chemical Engineering, Ningxia University, Yinchuan 750021, China; fll@nxu.edu.cn
4 Department of Chemical Engineering, Indian Institute of Technology Guwahati, Assam 781039, India; vkatiyar@iitg.ernet.in
* Correspondence: emwoo@mail.ncku.edu.tw; Tel.: +886-6-275-7575 (ext. 62670); Fax: +886-6-234-4496

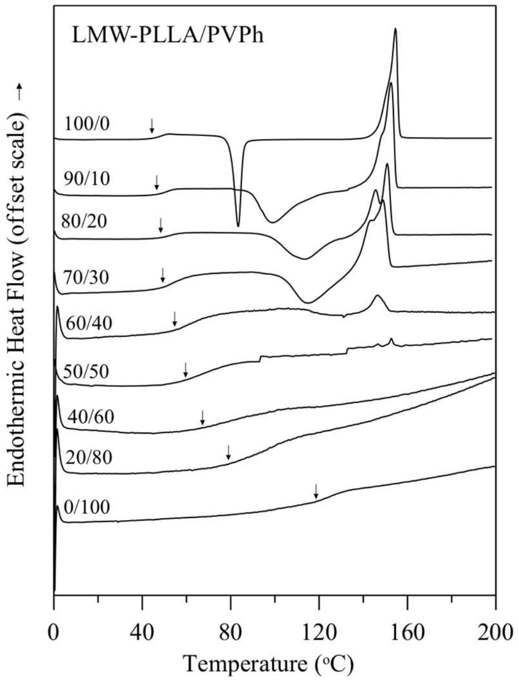

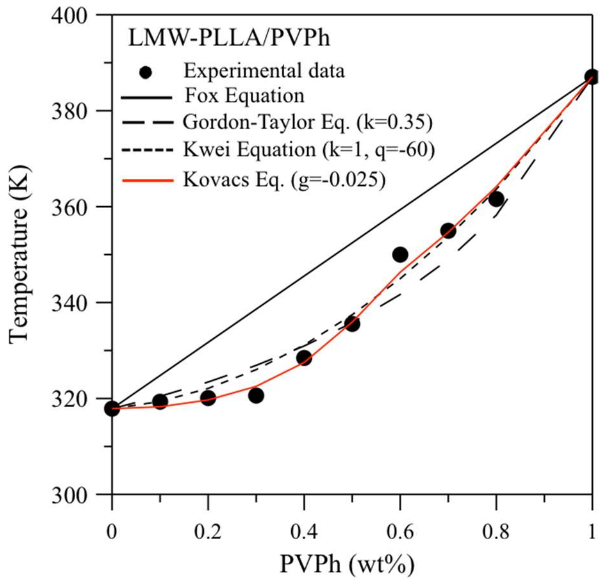

Figure S1. Phase diagrams for LMW-PLLA/PVPh blend: (**Top**) DSC thermograms, and (**Bottom**) T_g-composition curve.

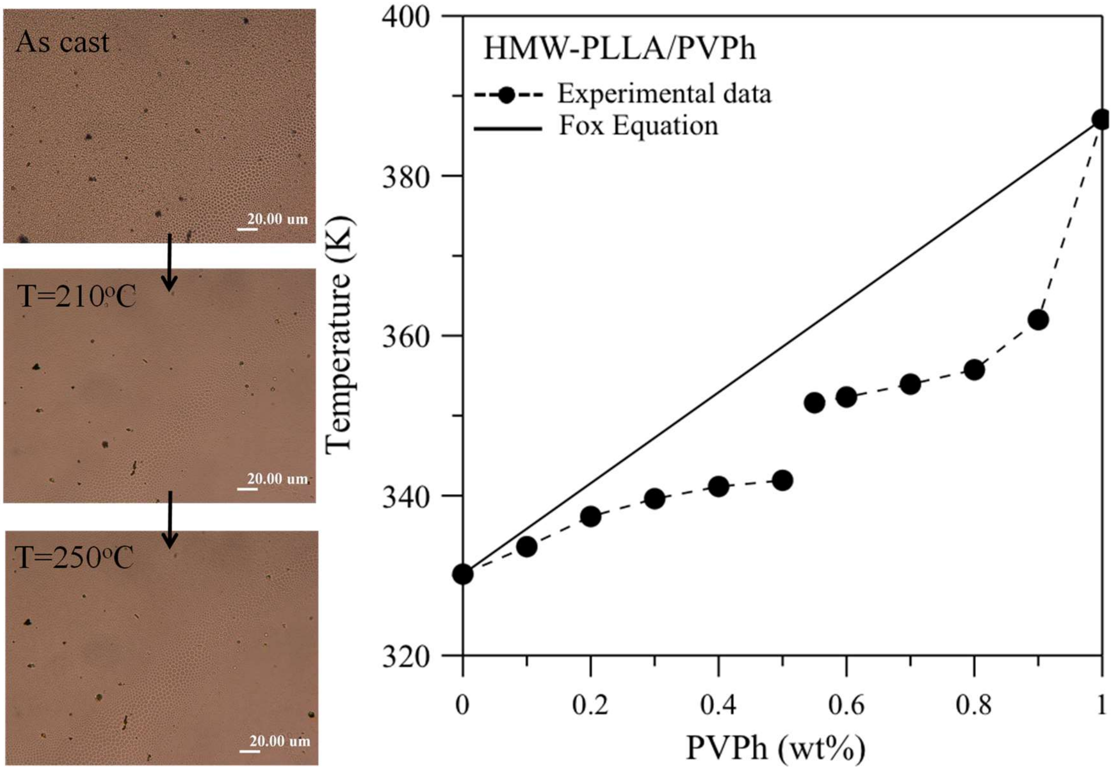

Figure S2. Phase diagrams for HMW-PLLA/PVPh blend: (**Left**) POM micrographs showing in-situ heating to above T_m; (**Right**) T_g vs. composition from DSC thermograms.

© 2018 by the authors. Submitted for possible open access publication under the terms and conditions of the Creative Commons Attribution (CC BY) license (http://creativecommons.org/licenses/by/4.0/).
